# Regulation of Cullin RING E3 Ubiquitin Ligases by CAND1 *In Vivo*

**DOI:** 10.1371/journal.pone.0016071

**Published:** 2011-01-13

**Authors:** Yee Shin Chua, Boon Kim Boh, Wanpen Ponyeam, Thilo Hagen

**Affiliations:** 1 Department of Biochemistry, Yong Loo Lin School of Medicine, National University of Singapore, Singapore, Singapore; 2 NUS Graduate School for Integrative Sciences and Engineering, Singapore, Singapore; University of Minnesota, United States of America

## Abstract

Cullin RING ligases are multi-subunit complexes consisting of a cullin protein which forms a scaffold onto which the RING protein Rbx1/2 and substrate receptor subunits assemble. CAND1, which binds to cullins that are not conjugated with Nedd8 and not associated with substrate receptors, has been shown to function as a positive regulator of Cullin ligases *in vivo*. Two models have been proposed to explain this requirement: (i) CAND1 sequesters cullin proteins and thus prevents autoubiquitination of substrate receptors, and (ii) CAND1 is required to promote the exchange of bound substrate receptors. Using mammalian cells, we show that CAND1 is predominantly cytoplasmically localized and that cullins are the major CAND1 interacting proteins. However, only small amounts of CAND1 bind to Cul1 in cells, despite low basal levels of Cul1 neddylation and approximately equal cytoplasmic endogenous protein concentrations of CAND1 and Cul1. Compared to F-box protein substrate receptors, binding of CAND1 to Cul1 *in vivo* is weak. Furthermore, preventing binding of F-box substrate receptors to Cul1 does not increase CAND1 binding. In conclusion, our study suggests that CAND1 does not function by sequestering cullins *in vivo* to prevent substrate receptor autoubiquitination and is likely to regulate cullin RING ligase activity via alternative mechanisms.

## Introduction

Cullin RING ligases are the largest family of cellular E3 ubiquitin ligases and control the stability of numerous cellular substrates involved in the regulation of the cell cycle, transcription and cell signaling. Cullin RING ligases are composed of one of 7 cullin homologues (in humans) which form a scaffold onto which the RING domain containing protein Rbx1/Rbx2 assembles at the cullin C-terminus [1], [2]. At the N-terminus, cullin proteins bind substrate receptor subunits, usually via an adaptor protein. For instance, Cullin1 (Cul1) forms SCF (Skp1-Cul1-F-box) complexes, in which Cul1 binds substrate receptors with a conserved F-box via the adaptor protein Skp1. All F-box proteins have different substrate binding domains which recruit ubiquitin ligase substrates, usually in a manner dependent on substrate phosphorylation or other posttranslational modifications. All cullin RING ligases require the modification with the ubiquitin like protein Nedd8 at a conserved lysine residue at the cullin C-terminus for full activity. Cullin neddylation is mediated by the Nedd8 specific APP-BP1/Uba3 E1 activating and Ubc12 E2 conjugating enzymes and is reversible via the action of the COP9 signalosome (CSN) [3], [4].

CAND1 is a cullin binding protein that only interacts with cullins that are unneddylated and are not associated with adaptor and substrate receptor subunits [5]–[8]. CAND1 is therefore believed to sequester cullin proteins in an inactive state [1], [2]. Nevertheless, loss of function studies in *Arabidopsis* have shown that CAND1 is required for cullin RING ligase function *in vivo* [9]–[11]. Thus, CAND1 mutant plants show distinct phenotypes and accumulation of cullin RING ligase substrates. Furthermore, a recent study in *C. elegans* provided evidence that CAND1 is required for the activity of a subset of Cullin RING E3 ligase complexes [12]. How CAND1 regulates cullin RING ligase activity is not well established. It has been hypothesized that by preventing binding of substrate receptors to cullin proteins, CAND1 prevents substrate receptor autoubiquitination in the absence of bound E3 ligase substrate [1], [2]. In support of this, CAND1 knockdown results in reduced cellular concentrations of the substrate receptor protein Skp2 [6], [13]. Alternatively, it has been proposed that cycles of CAND1 binding to and dissociation from cullins promote substrate receptor exchange and binding of substrate receptor subunits with lower affinity to Cul1 [14], [15]. However, the exact mechanisms through which CAND1 regulates cullin RING ligases remain to be identified. In this study, we investigated the role of CAND1 *in vivo*. Our results argue against a role of CAND1 in sequestering cullin proteins and preventing substrate receptor autoubiquitination in mammalian cells. Our data also suggest that the CAND1-Cul1 interaction is likely to be highly regulated via mechanisms that are independent of cullin neddylation and adaptor and substrate receptor subunit binding.

## Methods

### Plasmid constructs, mutagenesis and transfection of HEK293 cells

HEK293 cells were obtained from ATCC. Human CAND1 cDNA, a kind gift from Dr. Jong-Bok Yoon (Yonsei University, Korea), was PCR amplified using oligonucleotides which contained KpnI and XbaI sites at the 5′ and 3′ends, respectively, and the sequence encoding for a V5 tag at the 3′end and cloned into pcDNA3. For doubly tagged CAND1 constructs we used the same strategy and inserted the PCR products into modified pcDNA3.1 or pcDNA4/TO including a 5′ FLAG tag. All other plasmids were as previously described [13], [16]. Mutagenesis to generate the K472E/R473E mutant Cul1 was carried out using the Stratagene site-directed mutagenesis kit.

The T-Rex system (Invitrogen) was used to generate cell lines with tetracycline-inducible expression of FLAG-CAND1-HA and dominant-negative Cul1-V5 (dnCul1) and dominant-negative Ubc12-HA (dnUbc12) according to the manufacturer's instructions, as previously described [13], [16]. For DNA transfections, sub-confluent T-Rex-293 cells (Invitrogen) were transfected using Genejuice (Novagen) according to the manufacturer's instructions. For transfection of recombinant protein, TurboFect (Fermentas) was used according to the manufacturer's instructions. To validate the method, we transfected cells with recombinant GST fusion protein in the presence or absence of the protein transfection agent for two hours, followed by immunofluorescence staining for GST. It was observed that strong intracellular GST staining in approximately 50% of the cells was detectable, while no signal was present when no transfection agent was included.

### siRNA-mediated gene silencing

For siRNA transfections, RNAi Max Lipofectamine (Invitrogen) was used as transfection agent according to the manufacturer's instructions with the following annealed Silencer predesigned siRNA duplexes (Ambion) at a final concentrations of 20 nM: CAND1: siRNA ID 27001 (CAND1 siRNA#1), 140585 (CAND1 siRNA#2), 27093 (CAND1 siRNA#3); CSN5: 214069 (CSN5 siRNA#1); Negative controls: Silencer Negative Control siRNA #2. Cells were lysed three days after siRNA transfections for Western blot analysis.

### Immunoblotting

For immunoblotting, cells were washed with ice-cold PBS and then lysed in triton X-100 containing lysis buffer, as previously described [17]. Lysates were pre-cleared by centrifugation before use for Western blotting. Equal amounts of protein were loaded for Western blot analysis. The following antibodies were used: monoclonal anti-p27 (610241; BD Biosciences), monoclonal anti-Skp1 (H-6) (sc5281; Santa Cruz Biotechnology), goat polyclonal anti-Skp2 (N-19) (sc1567; Santa Cruz Biotechnology), rabbit polyclonal anti-CSN5 (ab12323; Abcam Ltd.), rabbit polyclonal anti-Cul1 (40990547; Zymed Laboratories), goat polyclonal anti-CAND1 (A-13) (sc-10672; Santa Cruz Biotechnology), goat polyclonal anti-GST (27-4577-01; GE Healthcare), rabbit polyclonal anti-PARP (9542, Cell Signaling Technology), monoclonal anti-GAPDH (G8140-04; US Biological), monoclonal anti-α-tubulin (236–10501; Molecular Probes), monoclonal anti-V5 (Serotec), monoclonal anti-FLAG M2 (Sigma), rat monoclonal anti-HA (clone 3F10) (Roche).

### Immunoprecipitation

10 µl of Anti-FLAG M2 agarose (Sigma) or 2.5 µg of V5 antibody, coupled to 10 µl of protein G-sepharose (Amersham Biosciences) was used for immunoprecipitations. 500 µl pre-cleared lysate from HEK293 cells transfected in 60 mm tissue culture plates was added. The samples were tumbled at 4°C for 2 hours and the agarose or sepharose beads were then washed four times in 1 ml of cold buffer containing 50 mM Tris (pH 7.5), 0.5% NP40, 5% glycerol, 0.5 mM EDTA, 50 mM NaCl and once in buffer containing 50 mM Tris (pH 7.5). The immunoprecipitated proteins were then denatured in SDS-sample buffer and subjected to SDS-PAGE and Western blotting.

### Immunofluorescence staining

For immunocytochemistry, cells were fixed using paraformaldehyde and, after permeabilization of cells with 0.1% triton X-100 and blocking with 5% normal goat serum, incubated with FLAG antibody and secondary TRITC-conjugated anti-mouse IgG. Nuclei were labelled using DAPI.

### Preparation of nuclear and cytoplasmic protein fractions

HEK293 cells were lysed in hypotonic lysis buffer (containing 10 mM Tris, pH 7.5, 10 mM KCl, 1 mM EDTA, 1 mM EGTA, 0.1% β-mercaptoethanol and protease inhibitor cocktail (Roche)). After incubation on ice for 20 min, cell lysates were subjected to a freeze-thaw cycle and then centrifuged at 3,000 rpm. The supernatant was used as cytoplasmic fraction. The pellet was washed three times in phosphate-buffered saline followed by extraction of nuclear proteins in high salt buffer (containing 20 mM Tris, pH 7.5, 420 mM NaCl, 1 mM EDTA, 1 mM EGTA, 25% glycerol, 0.1% β-mercaptoethanol and protease inhibitor cocktail (Roche)).

## Results

### Cullin proteins are the major CAND1 binding proteins

**Figure 1:**
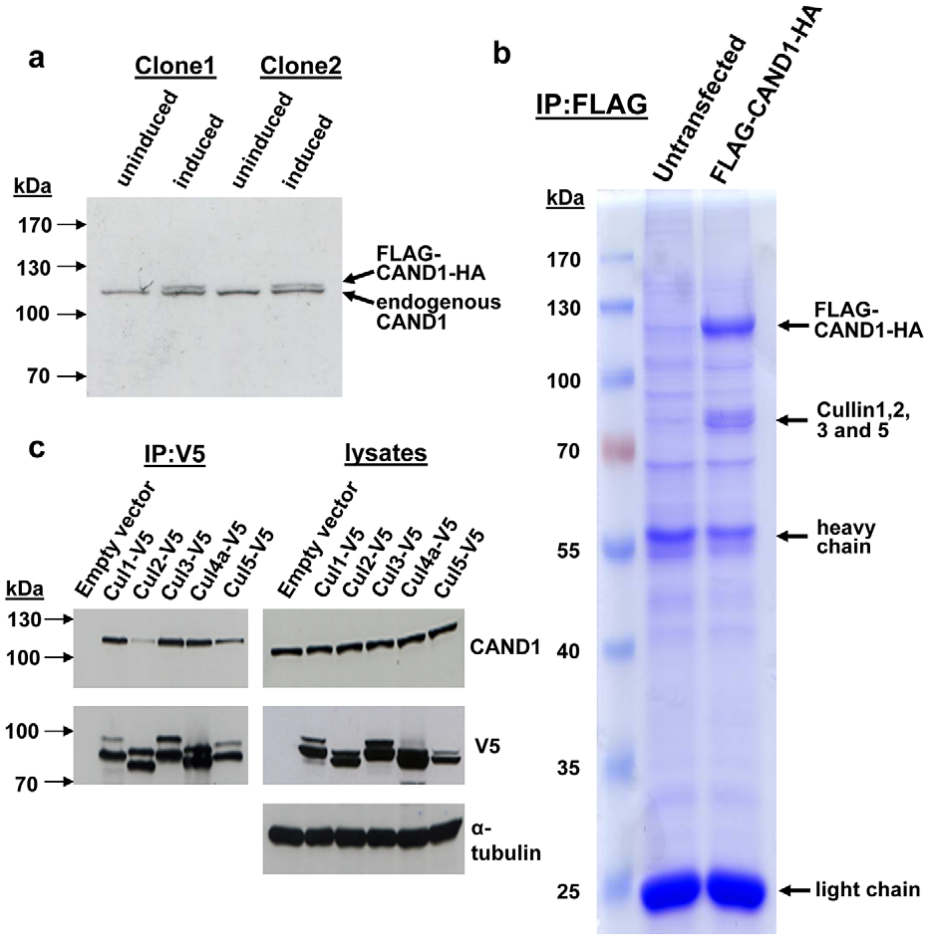
Cullin proteins are the major CAND1 binding proteins. (**a**) Western blot analysis of two different clones of HEK293 cells with stable expression of N-terminally FLAG-tagged and C-terminally HA-tagged CAND1 under a tetracycline-inducible promoter. Western blotting with CAND1 antibody indicates that the stably transfected CAND1 is expressed at physiological concentrations. (**b**) Cells harboring the stably transfected CAND1 were induced with 1 µg/ml of tetracycline for 24 hours. Control HEK 293 cells and tetracycline-induced cells were harvested and lysed with hypotonic lysis buffer consisting of 25 mM Tris, 2 mM EDTA, 2 mM EGTA and complete protease inhibitor cocktail tablet (Roche). Lysates were subjected to immunoprecipitation using anti-FLAG antibody to immunoprecipitate FLAG-CAND1-HA, immunoprecipitates were washed four times with 1X phosphate buffer saline (1X PBS) and analyzed by SDS-PAGE and Coomassie blue staining. A number of bands with a molecular weight of around 85 kDa were detected that were not present in the control. Mass spectrometric analysis of these bands revealed their identity as Cul1 (score:173; 12 detected peptides), Cul2 (score: 200; 17 detected peptides), Cul3 (score:63; 2 detected peptides), and Cul5 (score:47; 2 detected peptides). (**c**) HEK293 cells were transfected in 60-mm cell culture plates for two days with V5-tagged expression constructs for the cullin homologs indicated at the top of each panel. The cells were lysed, and the lysates were subjected to V5 immunoprecipitation, as described under Methods. Immunoprecipitates and aliquots of the cell lysates were analyzed by Western blotting with the indicated antibodies.

To identify CAND1 interacting proteins, we generated a HEK293 cell line with stable expression of N-terminally FLAG-tagged CAND1 under a tetracycline-inducible promoter. As shown in Fig. 1a, the FLAG-CAND1 in maximally induced cells was slightly less abundant than endogenous CAND1 and hence expressed at physiological concentrations. Cell lysates from induced and control cells were used for FLAG-immunoaffinity purification. When using triton X-100 containing lysis buffer, no interacting proteins could be detected in Coomassie Blue stained or silver stained SDS gels (data not shown). We then used hypotonic lysis buffer to break the cells and keep weaker protein-protein interactions intact. Using this approach, we detected a number of bands with a molecular weight of around 85 kDa that were not present in the control. Mass spectrometric analysis of these bands revealed their identity as Cul1, Cul2, Cul3, and Cul5. In addition to the cullin proteins, no other bands were detected that were specific for the FLAG-CAND1 induced cells. This suggests that cullin proteins are the major CAND1 interacting proteins. The absence of Cul4a and Cul4b is most likely related to their nuclear localization and the use of hypotonic lysis buffer with which nuclear proteins are not extracted. The results also suggest that a significant amount of CAND1 (and cullin proteins) is localized in the cytoplasm (see below).

We next used coimmunoprecipitation to compare the interaction of CAND1 with the various cullin protein homologues. To this end, cells were transfected with C-terminally V5-tagged cullins or empty vector and cell lysates subjected to V5 immunoprecipitation. Analysis of the V5 immunoprecipitates with CAND1 antibody revealed specific binding of endogenous CAND1 to all cullin proteins, although the interaction with Cul5 was somewhat weaker and the interaction with Cul2 markedly reduced compared to Cul1, Cul3 and Cul4a (Fig. 1c). In our previous study we found that CAND1 interacts primarily with Cul1 [16]. However, here we used NP-40 containing buffer in contrast to triton X-100 buffer (which was used in the previous study) to wash the immunoprecipitates and also detected significant interactions with other cullins, as shown in Fig. 1c. In further experiments, we focused on the interaction between Cul1 and CAND1 for which strong binding was detected.

### Cul1 neddylation regulates the interaction between Cul1 and CAND1 *in vivo*

**Figure 2:**
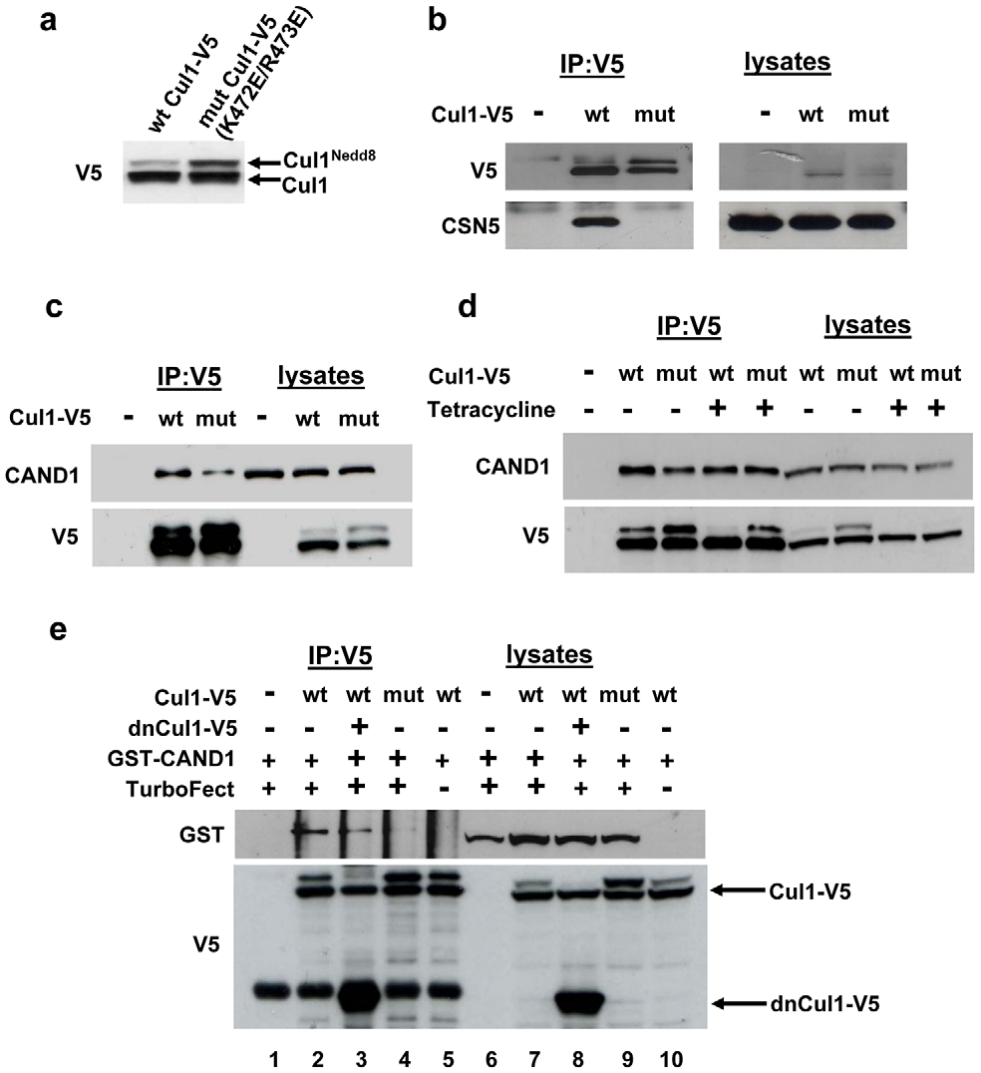
Neddylation regulates the interaction between Cul1 and CAND1 *in vivo*. (**a**) HEK293 cells were transfected with wild type or K472E/R473E mutant Cul1-V5, followed by Western blotting with V5 antibody. (**b–d**) Lysates from cells transfected with wild type or mutant (K472E/R473E) Cul1-V5 were subjected to immunoprecipitation using V5 antibody followed by Western blotting of lysates and immunoprecipitates with the indicated antibodies. In (**d**) dnUbc12 tet-on cells were used for transfection and induced with 1 µg/ml tetracycline for 24 hours prior to cell lysis in order to block Cul1 neddylation. (**e**) Cells were transfected in 60 mm dishes with 1 µg wild type or K472E/R473E mutant Cul1-V5 and 1.5 µg dnCul1-V5 or empty vector, as indicated. After two days, all plates were transfected with 10 µg of recombinant GST-CAND1 in the presence or absence of TurboFect protein transfection reagent. One hour after protein transfection, cells were rinsed and lysed and cell lysates subjected to V5 immunoprecipitation. The immunoprecioitates were analyzed by Western blotting with GST and V5 antibodies. As expected, no GST-CAND1 was observed in cell lysate and V5 immunoprecipitates when no protein transfection agent was included (see lanes 5 and 10). When GST was transfected into cells as a negative control, no binding to Cul1 could be observed (not shown).

CAND1 is known to bind to unneddylated cullin proteins [5], [6], [18]. To confirm that neddylation indeed regulates binding of CAND1 *in vivo*, we generated a hyperneddylation mutant of mouse Cul1 by mutating both Lys-472 and Arg-473 to Glu [5], [19]–[21]. K472E/R473E mutant Cul1 displayed a significantly increased level of Nedd8 modification (Fig. 2a). We hypothesized that the increased neddylation of K472E/R473E mutant Cul1 is due to reduced binding of the deneddylating CSN complex. We therefore measured the binding of wild type and K472E/R473E mutant Cul1 to the CSN5 subunit of the COP9 signalosome (CSN). As shown in Fig. 2b, in contrast to wild type Cul1, no interaction of CSN5 with K472E/R473E Cul1 could be detected, suggesting that the increased neddylation of the Cul1 mutant is due to reduced binding of CSN.

The K472E/R473E mutant Cul1 was then used to measure binding of CAND1 by coimmunoprecipitation. Binding of endogenous CAND1 to mutant Cul1 was significantly reduced compared to wild type Cul1 (Fig. 2c). This result suggests that increased Cul1 neddylation *in vivo* results in decreased CAND1 binding. Although we found in Fig. 2b that the increased neddylation of K472E/R473E mutant Cul1 is due to reduced CSN binding, it would also be possible that the increased neddylation of the mutant is a consequence of reduced CAND1 binding, possibly due to a conformational change in the Cul1 protein. To rule out this possibility, neddylation of wild type and K472E/R473E mutant Cul1 was inhibited. This was achieved by using a cell line which expresses a tetracycline inducible dominant negative Ubc12-HA (dnUbc12) (Fig. 2d) [13], [22]. Induction of dnUbc12 led to markedly reduced neddylation (compare lanes 8 and 9 with lanes 6 and 7 in Fig. 2d), although some neddylation was still observed in the immunoprecipitated wild type and mutant Cul1. When neddylation was inhibited, CAND1 binding to the mutant Cul1 was no longer reduced compared to wild type. This suggests that the K472E/R473E mutation does not interfere with CAND1 binding per se, but that increased neddylation *in vivo* reduces the CAND1-Cul1 interaction.

In order to further confirm these immunoprecipitation results, which measure steady state interactions between Cul1 and CAND1 *in vivo*, we used transfection of recombinant GST-CAND1 protein into cells and measured binding to Cul1 one hour after transfection using immunoprecipitation of Cul1-V5. As shown in Fig. 2e, recombinant GST-CAND1 protein was only present in cell lysates and V5 immunoprecipitates when the protein transfection agent was included. Within one hour of protein transfection, markedly less GST-CAND1 bound to K472E/R473E mutant Cul1 compared to wild type Cul1 (compare lanes 4 and 2), thus confirming that increased neddylation of Cul1 inhibits CAND1 binding *in vivo*.

### CAND1 is unlikely to function to sequester all inactive cullin proteins

**Figure 3:**
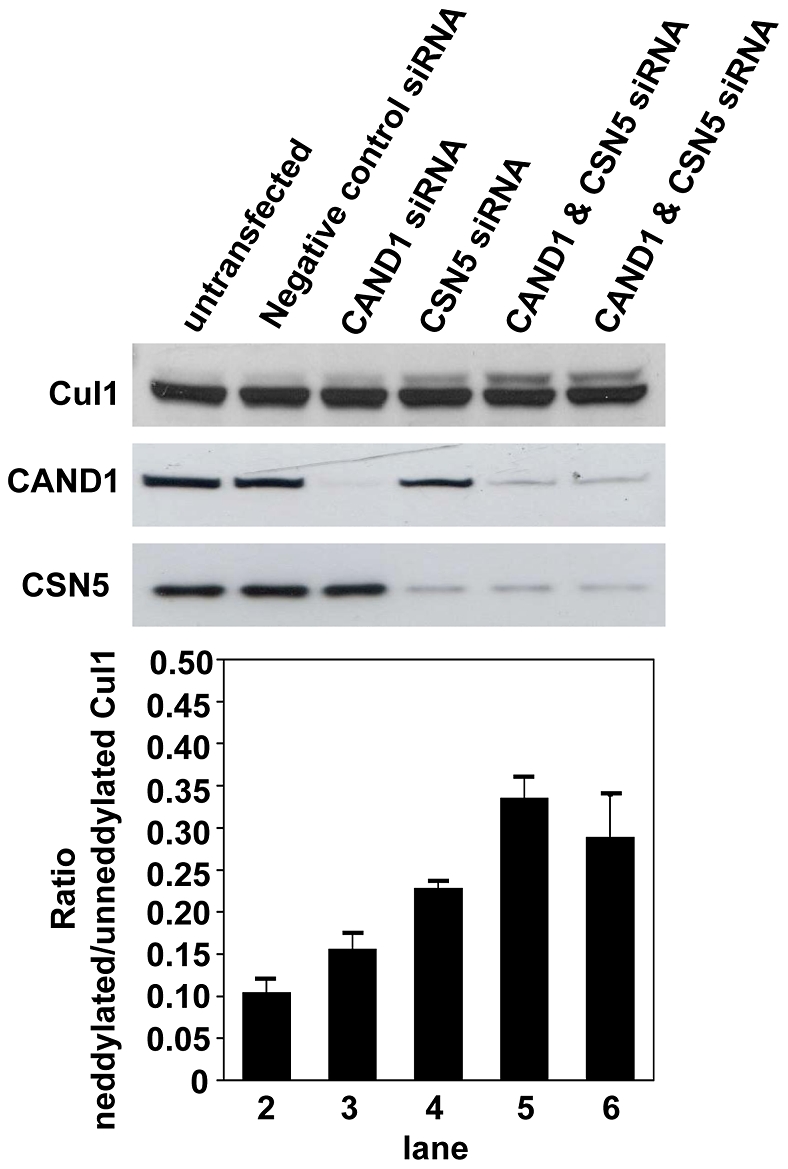
Effect of CAND1 and CSN5 knockdown on Cul1 neddylation. Cells were transfected with siRNA targeting CAND1 or CSN5 for three days, as described under Methods. In lanes 2 to 4, 20 nM of individual siRNA was transfected. In lane 5, 10 nM of each CAND1 and CSN5 siRNA and in lane 6, 20 nM of both siRNAs was used. Cul1 neddylation was assessed by Western blotting with Cul1 antibody. Densitometry analysis of the ratio of neddylated to unneddylated Cul1 is presented in the lower panel which is derived from two independent experiments which showed very similar trends.

The majority of cellular Cul1 is normally in the unneddylated form which may be due to sequestration by CAND1. However, siRNA-mediated knockdown of CAND1, which led to more than 90% CAND1 protein reduction, caused only a marginal increase in Cul1 neddylation (Fig. 3). This result suggests that the low basal neddylation of cullins is not due to sequestration by CAND1. It is possible that an increase in neddylation cannot be observed because of high deneddylating activity of CSN. We therefore also knocked down CSN5, which resulted in a greater increase in Cul1 neddylation. When CAND1 siRNA was cotransfected with CSN5 siRNA, an additive effect on Cul1 neddylation was observed. These results suggest that CAND1 may play a role in sequestering a certain amount, although not the majority of cellular Cul1.

**Figure 4:**
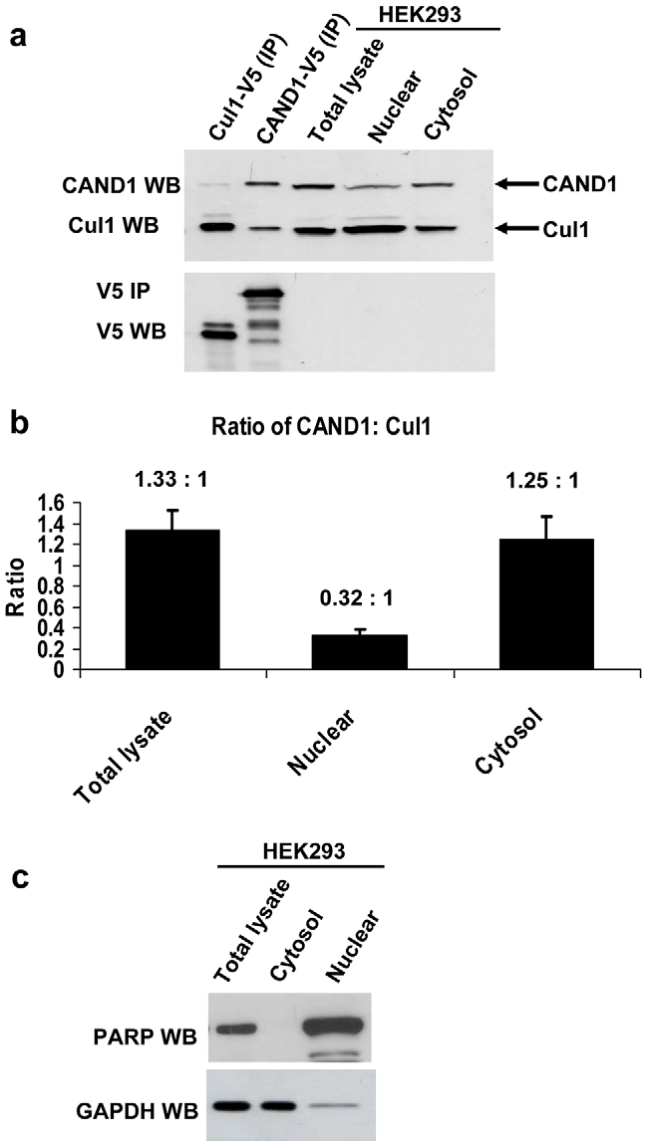
Cellular ratio of endogenous CAND1 and Cul1 proteins. (**a,b**) To compare cellular CAND1 and Cul1 protein expression, transfected and immunoprecipitated Cul1-V5 (lane 1 and CAND1-V5 (lane 2) were used as protein standards. Endogenous amounts of CAND1 and Cul1 protein in total lysate, nuclear and cytoplasmic fractions was measured in Western blots and compared to the respective protein standards by densitometry. Direct comparison of the two protein standards by Western blotting with V5 antibody (lower panel in (**a**)) allowed for calculation of the ratios of endogenous CAND1 to Cul1. Densitometry measurements from four independent experiments gave the means and S.E.M. values presented in (**b**). (**c**) The purity of the nuclear and cytoplasmic fractions, which were prepared as described under Methods, was confirmed by Western blotting with PARP and GAPDH antibodies.

To further test the hypothesis that CAND1 functions by binding and sequestering non-active cullin proteins, we determined if such a role of CAND1 would be compatible with the ratio of the endogenous cellular CAND1 and Cul1 proteins. To compare the relative expression of endogenous CAND1 and Cul1, we first transfected and immunoprecipitated the proteins from cell lysate using V5 antibody. The immunoprecpitated proteins, whose expression could be directly compared using detection of the V5 tag, served as protein standards in Western blots to normalize the endogenous cellular CAND1 and Cul1 amounts. For instance, as shown in the CAND1 and Cul1 Western blots in the upper panel of Fig. 4a, the CAND1 protein amount in the total HEK293 cell lysate (lane 3) was similar compared to the CAND1 protein standard (lane 2), whereas the Cul1 amount in the lysate (lane 3) was slightly less than the Cul1 protein standard (lane 1). Direct comparison of the CAND1 and Cul1 protein standards by V5 Western blot revealed similar amounts (lower panel in Fig. 4a). It therefore follows that the endogenous CAND1 concentration is slightly higher compared to the Cul1 concentration. This experiment was repeated four times in HEK293 cells and a ratio of CAND1 to Cul1 in total lysate of 1.33∶1 was determined after densitometry analysis (Fig. 4b). CAND1 and Cul1 were expressed at somewhat similar ratios in HCT116 cells (1.99∶1) and in HeLa cells (0.74∶1) (n = 2). We also separated HEK293 cells into nuclear and cytoplasmic fractions and the purity of these fractions was confirmed by Western blotting with PARP (nuclear marker) and GAPDH (cytoplasmic marker) antibodies (Fig. 4c). The measured ratios of CAND1 to Cul1 in the nucleus and cytoplasm were 0.32∶1 and 1.25∶1, respectively (Fig. 4a and b). Our results indicate that the endogenous concentrations of CAND1 and Cul1 in the cytoplasm are similar, while in the nucleus there is significantly less CAND1 compared to Cul1. Given that CAND1 interacts with various cullin homologues, it therefore appears unlikely that CAND1 can function to sequester and inactivate all free cullin proteins.

### CAND1 is predominantly a cytoplasmic protein in HEK293 cells

**Figure 5:**
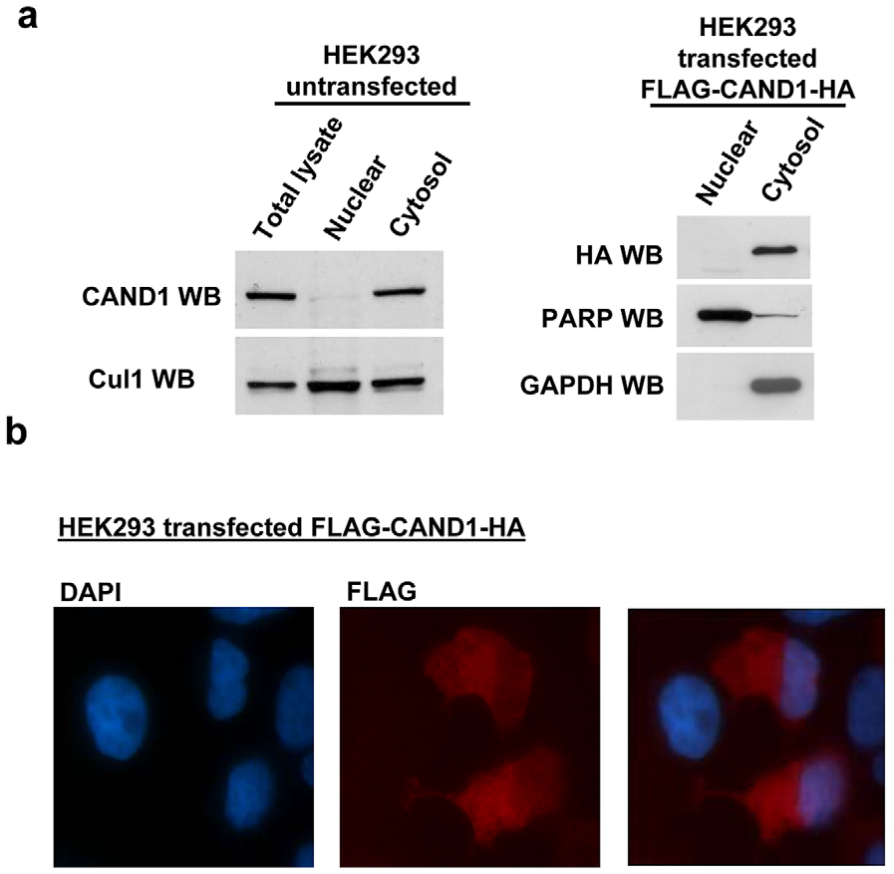
Nuclear and cytoplasmic distribution of CAND1. (**a**) In the left panel, untransfected HEK293 cells were fractionated into nuclear and cytoplasmic fractions as described under Methods. Equal amounts of total, nuclear and cytoplasmic proteins were loaded onto SDS gels and analyzed by Western blotting using CAND1 and Cul1 antibodies. The results shown are representative of three independent experiments. To quantify the ratio of nuclear versus cytoplasmic proteins, densitometry analysis was carried out and the results are mentioned in the text. In the right panel, cells were transiently transfected with FLAG-CAND1-HA, followed by preparation of nuclear and cytoplasmic fractions and Western blotting using the indicated antibodies. (**b**) Immunofluorescence staining of transfected HEK293 cells was carried out using FLAG antibody, as described under Methods.

The experiments in Fig. 4 indicate a threefold lower ratio of the CAND1 to Cul1 protein concentrations in the nucleus compared to that in the cytoplasm. The different ratios are mainly due to a 2.9 fold lower concentration of CAND1 in the nucleus compared to the cytoplasm [ratio of nuclear to cytoplasmic CAND1  = 0.34:1±0.09 (n = 3)] (Fig. 5a). In contrast, the nuclear and cytoplasmic concentration of Cul1 is approximately equal [ratio of nuclear to cytoplasmic  = 1.32:1±0.33 (n = 3)]. We also observed by Western blotting that transiently transfected FLAG-CAND1-HA was almost exclusively localized in the cytoplasm in HEK293 cells (Fig. 5a). Consistent with higher expression of CAND1 in the cytoplasm, stably transfected FLAG-CAND1-HA, stained with FLAG antibody, showed predominant cytoplasmic staining with very little staining in the nucleus (Fig. 5b). The immunofluorescence results with the transfected FLAG-CAND1-HA therefore substantiate the Western blot results for endogenous CAND1. The predominant presence of CAND1 in the cytoplasm suggests that the protein may regulate Cullin Ring ligases differentially dependent on their localization. It is also possible that the CAND1 subcellular localization is subject to regulatory events.

### Cul1 binds preferentially to substrate receptors

**Figure 6:**
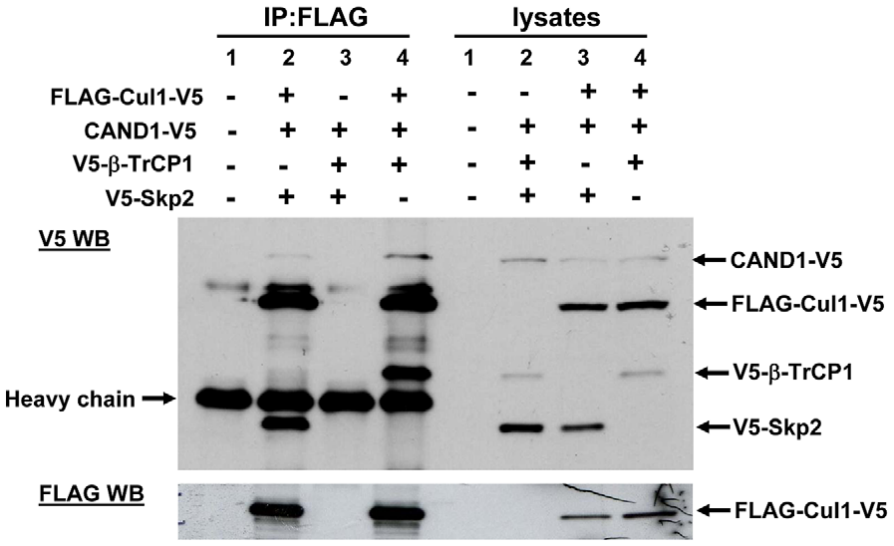
Cul1 binds preferentially to substrate receptors. HEK293 cells were cotransfected with the indicated plasmids. Cell lysates were used for immunoprecipitation with FLAG antibody followed by Western blotting.

Cul1 can bind to Skp1 adaptor and substrate receptor subunits and to CAND1 in a mutually exclusive manner [5]–[8]. To determine with which interaction partner Cul1 associates preferentially *in vivo*, we transfected cells with a plasmid encoding Cul1 carrying an N-terminal FLAG and a C-terminal V5 tag. The cells were co-transfected with V5-tagged CAND1 and β-TrCP or Skp2 substrate receptors. FLAG antibody was then used to immunoprecipiate Cul1 protein complexes. The FLAG-Cul1-V5 immunoprecipitates were analyzed in Western blots with V5 antibody to directly compare the amounts of Cul1, CAND1, β-TrCP and Skp2 in the Cul1 complex. Although CAND1 and β-TrCP were expressed at approximately equal amounts in the cell lysate, much more binding of β-TrCP to Cul1 was observed compared to CAND1 (see lane 4 of the total cell lysates and lane 4 of the FLAG immunoprecipitates in Fig. 6). Transfected Skp2 was expressed at higher concentrations. When comparing the ratio of Skp2 in the FLAG-immunopreciptates to that in the lysate, a marked enrichment of Skp2 protein was seen in the immunoprecipitates compared to CAND1 (compare the rations of Skp2 and CAND1 between lane 2 of the FLAG immunoprecipitates and lane 3 of the lysates). The enrichment of Skp2 in the immunoprecipiates compared to lysate was smaller than that observed for β-TrCP, which is likely due to the higher basal expression of Skp2 and saturation of Cul1 binding sites. When CAND1, β-TrCP and Skp2 were transfected in the absence of Cul1, none of the proteins was detected in the FLAG immunoprecipitates, confirming the specificity of the assay (see lane 2 of the lysates and lane 3 of the FLAG immunoprecipitates). Given the predominant localization of CAND1 in the cytoplasm, we also performed analogous experiments using cytoplasmic cellular fractions. Similarly to the total cell lysate, strong binding of both β-TrCP and Skp2 to Cul1 was observed while specific binding of CAND1 to Cul1 was low or undetectable (data not shown). Taken together, the results in Fig. 6 suggest that under *in vivo* conditions the substrate receptor proteins bind much stronger to Cul1 compared to CAND1. Our results are consistent with data published by Bornstein et al. [23], who showed that Skp2–Skp1 promotes the dissociation of CAND1 from Cul1 *in vitro*.

### Inhibiting binding of adaptor and substrate receptor subunits to Cul1 fails to increase CAND1 binding

**Figure 7:**
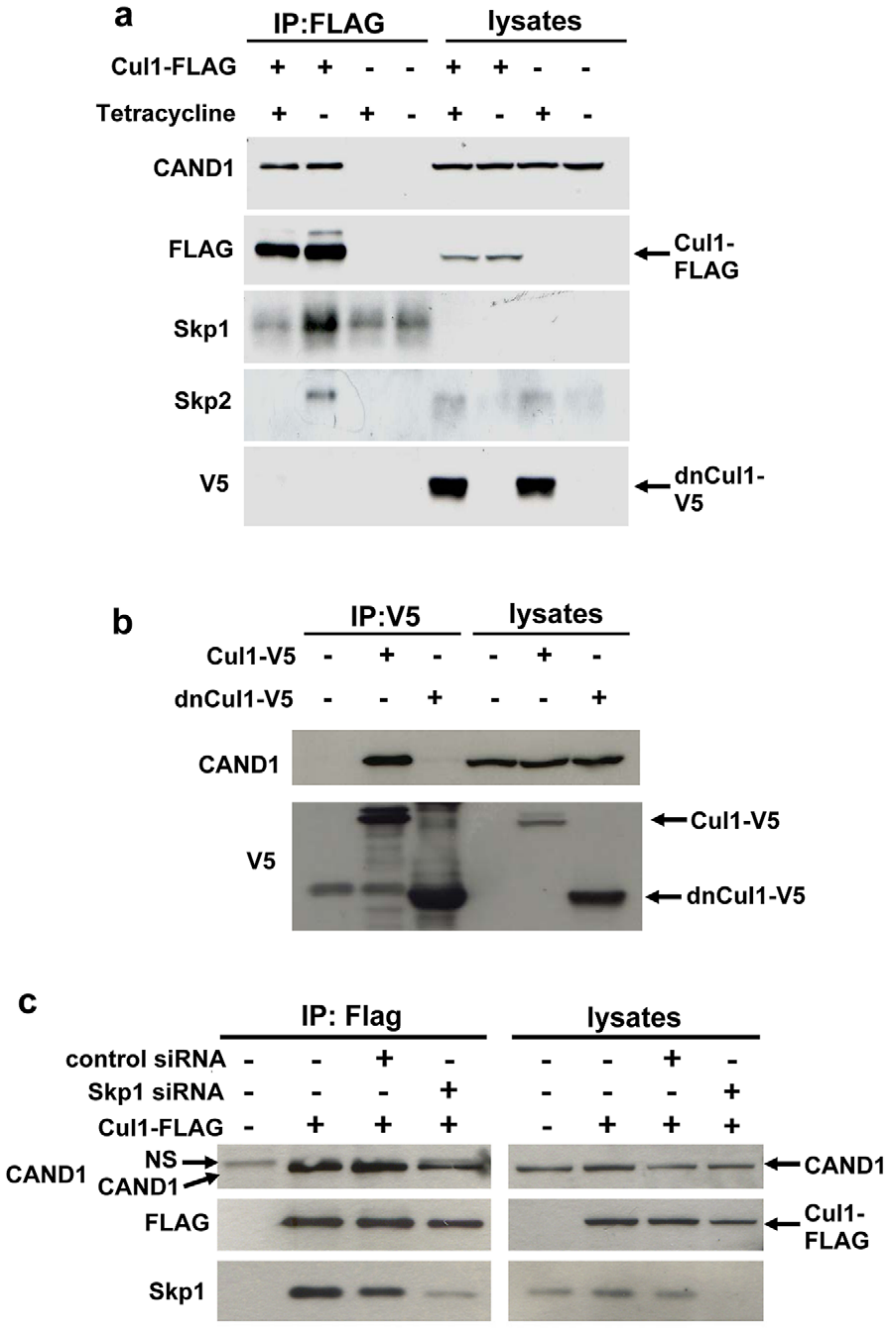
Inhibiting binding of substrate receptors fails to increase CAND1 binding. (**a**) dnCul1 tet-on cells were transfected with full length Cul1-FLAG as indicated and dnCul1-V5 expression was induced by adding 1 µg/ml tetracycline during the last 24 hours before cell lysis. Cell lysates were subjected to FLAG immunoprecipitation and Western blotting with the indicated antibodies. (**b**) Cells were transfected with full length Cul1-V5 or dnCul1-V5, as indicated, followed by V5 immunoprecipitation and Western blotting of immunoprecipitates and cell lysates with CAND1 and V5 antibodies. (**c**) Cells were transfected for three days with negative control or Skp1 siRNA (20 nM) and for the last two days with Cul1-FLAG plasmid as indicated. FLAG immunoprecpitation was then carried out followed by Western blotting with the indicated antibodies. The band labeled with NS in the CAND1 blot corresponds to a non-specific band.

If CAND1 were to function by sequestering inactive cullin proteins, it would be expected that CAND1 association with Cul1 increases when binding of Skp1 and substrate receptor proteins is prevented. To test this prediction, we used a stably transfected cell line with tetracycline inducible expression of dominant-negative Cul1 (dnCul1) [16]. dnCul1 lacks the C-terminus and is therefore unable to interact with CAND1. When induced, dnCul1 competes with full length Cul1 for binding to Skp1 and substrate receptor subunits. Thus, as shown in Fig. 7a, binding of endogenous Skp1 and Skp2 to full length Cul1 is abolished after addition of tetracycline to cells for 24 hours. Because substrate binding is a requirement for efficient cullin neddylation [16], [23], full length Cul1 also exhibits markedly reduced conjugation with Nedd8. Despite dissociation of Skp1 adaptor and substrate receptor from full length Cul1 and reduced neddylation, CAND1 binding to full length Cul1 did not increase, but actually exhibited a slight decrease. We also ruled out that the effect of dnCul1 is due to direct interaction with CAND1. As shown in Fig. 7b, only full length Cul1, but not dnCul1, interacted with CAND1. Similarly to the *in vivo* experiment in Fig. 7a, when using transfection of recombinant GST-CAND1 protein for one hour, reduced binding of CAND1 was observed in the presence of dnCul1 to inhibit binding of substrate receptor subunits (Fig. 2e).

As an alternative approach to decrease Skp1 and substrate receptor subunit binding to Cul1, we used siRNA silencing to knock down Skp1. As shown in Fig. 7c, Skp1 in the cell lysate was undetectable in cells transfected with Skp1 siRNA, but not with negative control siRNA. Some Skp1 protein was still bound to Cul1 in the immunoprecipitates, although much less compared to untransfected or negative control siRNA transfected cells. Knockdown of Skp1 also did not increase CAND1 binding to Cul1 (Fig. 7c). Thus, taken together, these results provide further evidence that CAND1 does not function by binding and sequestering inactive cullin proteins, but suggests that CAND1 binding to cullin proteins *in vivo* is regulated via other mechanisms.

## Discussion

CAND1 is an important regulator of cullin RING ligases. Although the interaction of CAND1 with cullin proteins, in particular with Cul1, and its consequences have been well characterized in structural and *in vitro* studies [18], the exact role of CAND1 in regulating E3 ligase activity *in vivo* is still unknown [1], [2]. According to one proposed model, CAND1 functions by binding to and inactivating of cullin proteins in the absence of ubiquitination substrates. This is believed to prevent autoubiquitination of substrate receptor subunits. In a different model, dynamic association and dissociation cycles of CAND1 with cullin proteins promote the exchange of substrate receptor subunits. In this study, we present a number of findings which suggest that CAND1 is unlikely to sequester all inactive, non-substrate bound cullin proteins. Thus, the ratio of endogenous CAND1 and Cul1 protein is likely to be too low, especially in the nucleus, for CAND1 to associate with all cellular non-substrate bound cullin proteins. Furthermore, CAND1 binding to Cul1 *in vivo* is much weaker compared to binding of substrate receptors. It should be noted that one alternative explanation is also that the different apparent binding affinities of CAND1 and substrate receptors for Cul1 are a consequence of other factors, such as different cellular localization of CAND1 and substrate receptor subunits or binding to and sequestration by other proteins. Finally, we found that preventing binding of adaptor and substrate receptor subunits to Cul1 (by overexpressing dominant negative Cul1 or silencing Skp1) does not increase CAND1 binding. These results suggest that CAND1 binding to cullin proteins may be highly regulated via mechanisms that are different from competition with adaptor and substrate receptor subunits for cullin proteins.

Given that CAND1 binds only non-Nedd8 conjugated cullin proteins, cullin neddylation could be an important regulator of CAND1 binding. Indeed, we observed that increased neddylation of Cul1 *in vivo* in the K472E/R473E Cul1 mutant results in decreased CAND1 binding (see Fig. 2). The increased neddylation in the K472E/R473E Cul1 mutant, which leads to the disruption of a buried salt bridge in the four-helix bundle domain of Cul1 [24], is likely a result of reduced binding of the deneddylating CSN complex (see Fig. 2b). Indeed, the four-helix bundle has been reported to be required for binding of CSN to Cul1 [25]. Our results in Fig. 2d indicate that the K472E/R473E mutation per se does not inhibit CAND1 binding, but that decreased CAND1 binding is due to the increased neddylation levels. On the other hand, normal neddylation levels of all cullin proteins are relatively low, and yet, binding of CAND1 to Cul1 under basal conditions is weak. Furthermore, inhibiting neddylation by expressing a dominant negative form of Ubc12 does not increase CAND1 binding (data not shown). Thus, factors other than neddylation are important in the regulation of the CAND1-Cul1 interaction.

Given the limited role of substrate receptor binding and neddylation in regulating the CAND1-Cul1 association *in vivo*, it is likely that the interaction between the two proteins is regulated by other mechanisms. For instance, CAND1 binding to cullin proteins may be regulated by posttranslational modifications in either of the proteins or by other interacting proteins. CAND1 binding may also be directly coupled to substrate ubiquitination. Future *in vitro* and *in vivo* studies will be necessary to investigate these potential mechanisms and to understand how CAND1 regulates cullin RING E3 ligases.
